# MiR-320d Inhibits Progression of EGFR-Positive Colorectal Cancer by Targeting TUSC3

**DOI:** 10.3389/fgene.2021.738559

**Published:** 2021-10-18

**Authors:** Zhu Yufeng, Qi Ming, Wu Dandan

**Affiliations:** ^1^ Department of General Surgery, The First Affiliated Hospital of JinZhou Medical University, JinZhou, China; ^2^ Department of Ultrasound, The First Affiliated Hospital of JinZhou Medical University, JinZhou, China; ^3^ Department of Gastroenterology, The First Affiliated Hospital of JinZhou Medical University, JinZhou, China

**Keywords:** miR-320d, epidermal growth factor receptor, colorectal cancer, tumor-suppressor candidate 3, tumor

## Abstract

**Background:** The mechanism of miR-320d in EGFR-positive colorectal cancer (CRC) has not been fully elucidated. The aim of the present study was to explore the molecular mechanism of miR-320d in CRC.

**Methods:** The miRNA microarray analysis was conducted to identify differential expressed miRNAs. The expression of miR-320d was validated using quantitative real-time PCR. EGFR-positive CRC cells were transfected with miR-320d mimic and inhibitor, after which cell proliferation, migration, and invasion were assayed. The relationship between miR-320d and TUSC3 was confirmed using bioinformatics and dual-luciferase reporter gene assays. Proteins involved in signaling pathways and the epithelial–mesenchymal transition were detected with Western blot.

**Results:** We found that the miR-320d expression is associated with tumor size and distant metastasis in colorectal cancer. Overexpression of miR-320d in EGFR-positive HCT-116 and SW480 cells decreased not only the proliferation ability but also the invasion and migration ability. In addition, miR-320d had the ability to inhibit epithelial-to-mesenchymal transition. Luciferase assays revealed that miR-320d directly targets the 3′-UTR of TUSC3. TUSC3 was downregulated by miR-320d at both the protein and mRNA levels in EGFR-positive CRC cell lines.

**Conclusion:** Generally, our results demonstrated that miR-320d could inhibit the malignant phenotype of EGFR-positive CRC through targeting TUSC3. The miR-320d might be a potential therapeutic target for EGFR-positive CRC.

## Introduction

## Background

Colorectal cancer (CRC) is the second leading cause of cancer death worldwide (Bray et al., 2018). As one of the most common malignant gastrointestinal tumors, CRC develops rapidly, with high mortality rates (Jiang et al., 2017). Early-stage CRC can be ideally treated by radical surgical resection, while advanced CRC is usually treated by a combination of surgery and chemotherapy, but postoperative recurrence and uncontrollable metastasis of tumor cells are among the most important causes of death in CRC patients (Zhang et al., 2021). Therefore, it is important to explore useful prognostic biomarkers or therapeutic targets for CRC to ameliorate our shortcomings in disease diagnosis, prevention, and treatment.

The epidermal growth factor receptor (EGFR) is the product of the proto-oncogene CerbB-1, which has tyrosine kinase transmembrane activity and mediates gene expression in the nucleus, affecting cell growth and differentiation (Yang et al., 2016). EGFR is located on chromosome 7 and is widespread in most tissues and cells, and EGFR can directly affect cell proliferation and migration through the PI3K/AKT pathway and RAS/RAF/ErK pathway (Gharwan and Groninger, 2016). In CRC, EGFR is highly expressed and is closely related to the clinicopathological stage of the tumor, the extent of lymph node involvement, vascular infiltration, and tumor distant metastasis status (Lo Nigro et al., 2016). More than 90% of CRC patients overexpress EGFR, so it is necessary to further explore the molecular biological mechanism of EGFR-positive CRC and identify more precise biomarkers to improve the effectiveness of individualized treatment strategies (Morgan et al., 2018).

MicroRNAs are a class of endogenous noncoding small single-stranded RNAs of approximately 18–25 nucleotides in length, which are produced by DNA transcription and are not directly involved in encoding proteins. However, microRNAs can posttranscriptionally regulate the expression of target genes through the RNA-induced silencing complex (Yao et al., 2019). The expression of miRNAs is closely related to cell growth, proliferation, differentiation, and apoptosis, and 50% of miRNAs are related to the tumor-related gene (Akbari et al., 2021). In this study, we compared miRNA expressions between EGFR-positive primary CRC and paired adjacent normal tissue; microRNA microarray analysis and bioinformatics analysis were used to investigate the potential molecular mechanisms of EGFR-positive CRC, and miR-320d caught our attention.

MiR-320d is a member of the miR-320 family, and the serum miR-320d may be used to predict survival of rectal cancer patients (Mjelle et al., 2017). miR-320d may be CRT-sensitive microRNAs in CRC patients and may be used as sensitive biomarkers in CRC patients (Du et al., 2019). The microRNA-320 family is downregulated in colorectal adenoma and affects tumor proliferation by targeting CDK6 (Tadano et al., 2016). However, the molecular function of miR-320d in EGFR-positive CRC was unknown. The experimental validation is still lacking, and the potential mRNA targets of miR-320d and the regulatory mechanisms in EGFR-positive CRC remain unknown. In this study, we will investigate the function of miR-320d in EGFR-positive CRCs.

## Materials and Methods

### Patients and Samples

We obtained CRC samples and paired adjacent normal tissue samples of CRC patients from our hospital. The distance of paired adjacent normal tissue samples to cancer is larger than 7 cm. All tissues were stored immediately in liquid nitrogen and conserved at −80°C for further use. A portion of all the samples was subjected to pathological analysis performed by two independent, blinded, and experienced pathologists. Informed consent was obtained from CRC patients. No cancer patients received chemotherapy or radiotherapy before surgery. We first sent two paired samples to Wanlei Biotech (Shenyang, China), for miRNA expression microarray profiling. The first CRC patient was male, 54 years old; the tumor size was 2.5 cm, the tumor was poor-moderated differentiated tubular adenoma and with lymph node metastasis, and the TNM stage was pT_3_N_2a_. The second CRC patient was male, 49 years old; the tumor size was 3.5 cm, the tumor was poor-moderated differentiated tubular adenoma and with lymph node metastasis, and the TNM stage was pT_3_N_1_cM_1a_. Then, other tissues were used for validation study. This study was reviewed and approved by the Research Ethics Committee of Jinzhou Medical University (Jinzhou, Liaoning, PR China).

### miRNA Microarray Analysis

Using miRNA microarrays, we analyzed the CRC samples and the paired adjacent normal tissue samples. Total RNA was isolated from CRC tissue with a miRNeasy Mini Kit (Qiagen), following the manufacturer’s instructions. The protocol of Agilent Gene Chips and the raw data are available online (https://www.ncbi.nlm.nih.gov/geo/query/acc.cgi?acc=GSE62007). Then, we used the R software and limma package to process the data. The differential expression of miRNAs was assessed using the |logFC| > 2 and adjusting the *p* < 0.05 level.

### Cell Culture and Transfection

The human CRC cell lines SW480, SW620, HT29, and HCT116 were purchased from Wanleibio (Shenyang, China). Cells were inoculated in DMEM high-sugar medium and cultured at 37°C, saturated humidity, and 5% CO_2_ volume fraction (subsequent cell cultures were performed under these conditions). Logarithmic growth phase cells were collected to continue the experiment.

The cells were passaged in DMEM medium, and when the cell fusion reached 80%, CRC cells were inoculated into 24-well cell culture plates, and CRC cells were transfected according to Entranster™-R4000 instructions. 1) In the microRNA overexpression group, 100 nmol of miR-320d-mimic was added to each well; 2) in the microRNA inhibition group, 100 nmol of miR-320d-inhibitor was added to each well; and 3) in the negative control group: 100 nmol of miR-320d-NC was added to each well. The incubation was continued under the conditions described previously, and the dose of each treatment factor was based on the transfection instructions.

### RNA Extraction and Quantitative Real-Time PCR

The miRNA was extracted using TRIzol (Invitrogen), and the operation was performed strictly according to the kit instructions. The purified RNA was stored at −80°C for backup. The cDNA was synthesized by reverse transcription using HiScript^®^ II Reverse Transcriptase Kit; the reaction system was 20 μl, and the reaction conditions were 42°C for 3 min, 60°C for 15 min, and 85°C for 5 min. The cDNA was used as the template for qPCR using ChamQ universal SYBR qPCR Master Mix for qPCR; the reaction system was 20 μl, with the following reaction conditions: 95°C for 2 min, 40 cycles of 60°C for 5 s, and 95°C for 10 s. U6 was used as the internal reference, and the relative expression was expressed as 2-ΔΔCt. The experiment was repeated three times.

### Cell Proliferation Assay

CRC cells at the logarithmic growth stage were inoculated with 100 μl/well in a 96-well cell culture plate and transfected after 24 h of incubation. After 24, 48, and 72 h, 10 μl of MTT solution (5 mg/ml) was added to each well and incubated for 4 h. Then, 150 μl of dimethyl sulfoxide was added to each well and shaken for 10 min, and the absorbance value (A) was measured at 570 nm with an automatic enzyme marker.

### Transwell and Wound-Healing Assay

In the invasion assay, 40 μl of diluted Matrigel was applied to the Transwell. The concentration of the cell suspension was adjusted to 1×10^4^/ml. The transfected cells diluted with serum-free medium were added to the upper chamber of the Transwell, and the DMEM medium containing 10% FBS was added to the lower chamber. Three replicate wells were set up for each group. After 48 h incubation, the chambers were washed with PBS buffer, fixed in 95% alcohol, stained with 0.1% crystalline violet staining, rinsed, and air-dried. The number of perforated cells was observed under a microscope, and ImageJ software was used to calculate the number of cells penetrating the pores. The migration assay was performed in the Transwell chamber without the addition of Matrigel, and the other steps were the same as those for the invasion assay. For the wound healing assay, each group of cells was inoculated in six-well plates with 1×10^6^ cells per well and then incubated at 37°C, 5% CO_2_, and 100% relative humidity until about 100% density of cells. The cell monolayer was subsequently scratched with a 200-μl pipette tip and then washed off by PBS. The culture was then continued under the same conditions for 24 h. The monolayer images were observed using an inverted microscope and obtained.

### Western Blot Analysis

The transfected cells were collected and lysed on ice in RIPA lysis buffer with phosphatase and protease inhibitors followed by centrifugation at 14,000 rpm for 20 min at 4°C, and then the protein concentration was determined using the bicinchoninic acid (BCA) protein assay kit. The proteins were separated by electrophoresis on a 10% SDS-PAGE gel at 90 V for 120 min. The proteins were transferred to PVDF membrane and blocked in 5% fat-free milk for 2 h at room temperature then incubated with primary antibodies against TUSC3 (1:1,000, Abcam, ab230520), E-cadherin (1:10,000, Abcam, ab 40,772), vimentin (1:5,000, Abcam, ab92547), PI3K/AKT signaling pathway panel (Abcam, ab283852), β-actin (1:5,000, Abcam, ab6276), and GAPDH (1:5,000, Abcam, ab8245) at 4°C overnight. The full original pictures are shown in the supplementary file.

### The microRNA Target Prediction

Target gene prediction softwares, including TargetScan 7.2 (Agarwal et al., 2015), PITA(Paraskevopoulou et al., 2013), miRanda (Wang et al., 2014), and PicTar (Krek et al., 2005), were used to predict the potential targets of the microRNA. To elucidate the biological function of the target genes, we performed the pathway enrichment analysis by utilizing DAVID (http://david.abcc.ncifcrf.gov/). A *p*-value less than 0.05 and count more than 2 were selected as the cutoff criteria. The CRC samples in the TCGA database were divided into TUSC3 high-expression group and TUSC3 low-expression group according to the median TUSC3 expression, and GSEA software was used to analyze the possible mechanisms of TUSC3 involvement in CRC development by GSEA with the hallmark gene set as the reference gene set, and the runs of gene permutations were set to 1,000, and a significant enriched gene set was defined by nominal *p*-value and FDR q-value < 0.05.

### Luciferase Assay

The TUSC3 gene 3′UTR wild-type and mutant recombinant psiCHECK2 luciferase reporter vector were constructed, and the targeting relationship between miR-320d and TUSC3 was verified by the dual-luciferase reporter gene system. HCT116 cells were co-transfected with 200 nmol/l miR-320d and 100 ng plasmid, and cell lysates were collected in 24-well plates for 48 h. The fluorescence intensity of sea kidney and firefly luciferase was analyzed using the dual-luciferase reporter analysis system, and the final results were expressed as the ratio of firefly luciferase and sea kidney luciferase activities, and the experiments were repeated three times.

### Statistical Analysis

Student’s t-test was used to analyze differences between two groups. A one-way ANOVA was performed to detect statistical differences among multiple groups. All statistical analyses were performed using the SPSS 22.0 (SPSS, Chicago, IL). A two-tailed value of *p* < 0.05 was considered statistically significant.

## Result

### Differentially Expressed miRNAs in Epidermal Growth Factor Receptor-Positive Colorectal Cancer

**FIGURE 1:**
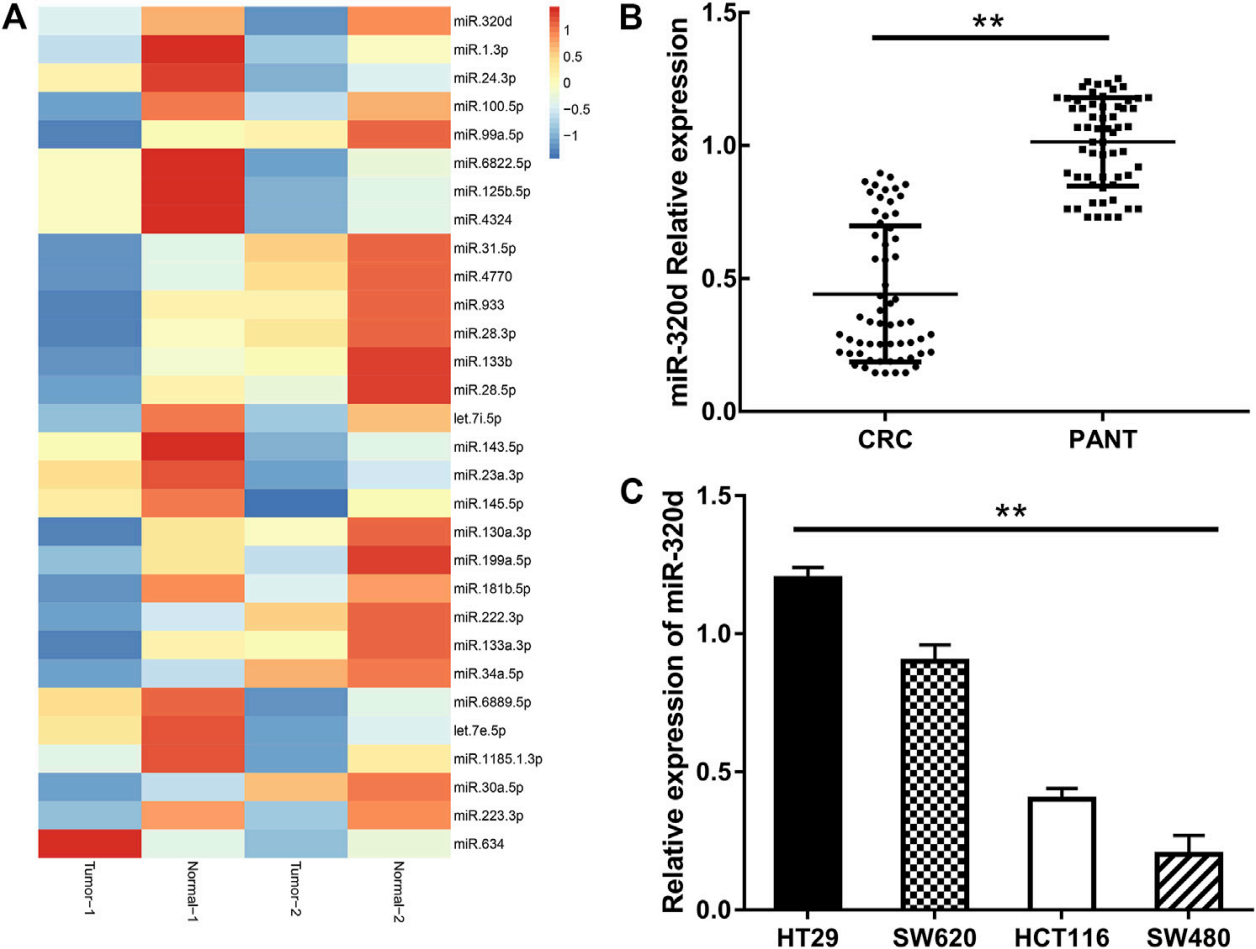
The expressions of miR-320d were examined in the EGFR-positive colorectal cancer and cell lines. **(A)** The heatmap of the top 30 differentially expressed microRNA in EGFR-CRC and paired adjacent normal tissue. Each column represents a sample, and each row represents the expression level of a given microRNA. The color scale represents the raw Z score ranging from blue (low expression) to red (high expression). **(B)** qRT-PCR analysis of miR-320d in EGFR-positive CRC tissues (*n* = 60) and adjacent normal tissues (*n* = 60). **(C)** qRT-PCR analysis of miR-320d in EGFR-positive CRC cell lines. Statistical analysis was conducted using Student’s t test. **p< 0.01.

We compared miRNA expressions between EGFR-positive primary tumors and paired adjacent normal tissue. A total of 139 differentially expressed miRNAs were identified, including 79 upregulated miRNAs and 60 downregulated miRNAs. We developed a heat map of the top 30 differentially expressed miRNAs, showing the significantly differential distribution of the miR-320d (Figure 1A). Combined with literature search, we select miR-320d for further study.

### MiR-320d Was Suppressed in Epidermal Growth Factor Receptor-Positive Colorectal Cancer Tissues

The expression levels of miR-320d in CRC tissues are much lower than those in the paired adjacent normal tissue (Figure 1B). We then examined the expression level of miR-320d in a series of EGFR-positive CRC lines; miR-320d was more lowly expressed in SW480 and HCT116 cell lines (Figure 1C). We next analyzed the correlation of miR-320d with clinicopathological characteristics, and the clinical features are shown in Table 1. The expression of miR-320d was significantly associated with distant metastasis, tumor size, and EGFR status, which means miR-320d might function as a tumor suppressor in EGFR-positive CRC.

**TABLE 1:** Relationship between miR-320d expression and CRC

Clinicopathological parameter	Number (%)				Low (30)	High (30)	*p* value
Sex
Female	26		(43.33%)		12	14	0.602
Male	34		(56.67%)		18	16	
Age (year)
<60	22		(36.67%)		13	9	0.284
≥60	38		(63.33%)		17	21	
Clinical stage
I	11		(18.33%)		6	5	0.493
II	17		(28.33%)		7	10	
III	22		(36.67%)		10	12	
IV	10		(16.67%)		7	3	
Lymph node metastasis
N0	33		(55.00%)		17	16	0.831
N1	16		(26.67%)		7	9	
N2	11		(18.33%)		6	5	
Distant metastasis
Absent	43		(71.67%)		17	26	**0.01**
Present	17		(28.33%)		13	4	
Histological differentiation
Well/moderate	30		(50.00%)		16	14	0.438
Poor	30		(50.00%)		13	17	
Tumor size
<5 cm	36		(60.00%)		14	22	**0.035**
≥5 cm	24		(40.00%)		16	8	
EGFR
Negative	27		(45.00%)		7	20	**0.001**
Positive	33		(55.00%)		23	10	

### Target Prediction of miR-320d

**FIGURE2:**
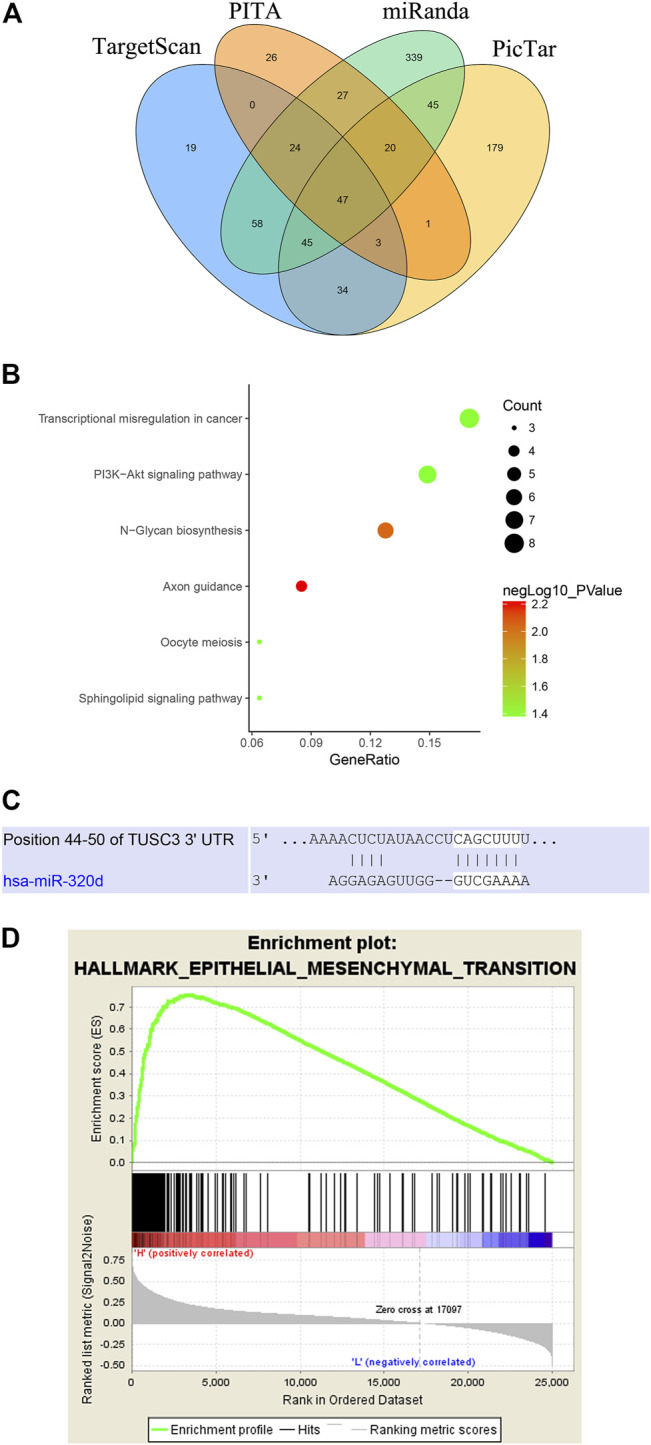
The bioinformatics of miR-320d in EGFR-positive colorectal cancer. **(A)** Venn diagrams of putative miR-320d targets predicted by TargetScan, miRanda, PicTar, and PITA. **(B)** The KEGG pathways that are enriched for the miR-320d targets. **(C)** The 3′-UTR of TUSC3 mRNA harbors one miR-320d cognate sites. **(D)** Performance of GSEA based on TCGA datasets.; Abbreviations: KEGG, Kyoto Encyclopedia of Genes and Genomes; GSEA, Gene Set Enrichment Analysis; TCGA, The Cancer Genome Atlas.

To explore the underlying mechanism of miR-320d, we used bioinformatics software (TargetScan, PicTar, PITA, and miRanda) and identified a total of 47 consensus target genes (Figure 2A). The overlap section of predicted target genes was collected for the following analysis. KEGG pathway analysis indicated that these consensus-targeted genes were mainly involved in N-glycan biosynthesis. Transcriptional misregulation in cancer and the PI3K-Akt signaling pathway, which were associated with tumor development, suggests that miR-320d may regulate CRC progression (Figure 2B). According to the sequence analysis of miR-320d, we found that miR-320d could be directly combined with the corresponding 3′UTR of TUSC3 in humans (Figure 2C). Then, we performed GSEA using TCGA data and found that the epithelial–mesenchymal transition (EMT) (NES = 2.25) was most commonly enriched in the TUSC3 high-expression group (nominal *p*-value < 0.05, FDR q-value<0.05) (Figure 2D).

### MiR-320d Inhibits Epidermal Growth Factor Receptor-Positive Colorectal Cancer Malignant Phenotypes

**FIGURE3:**
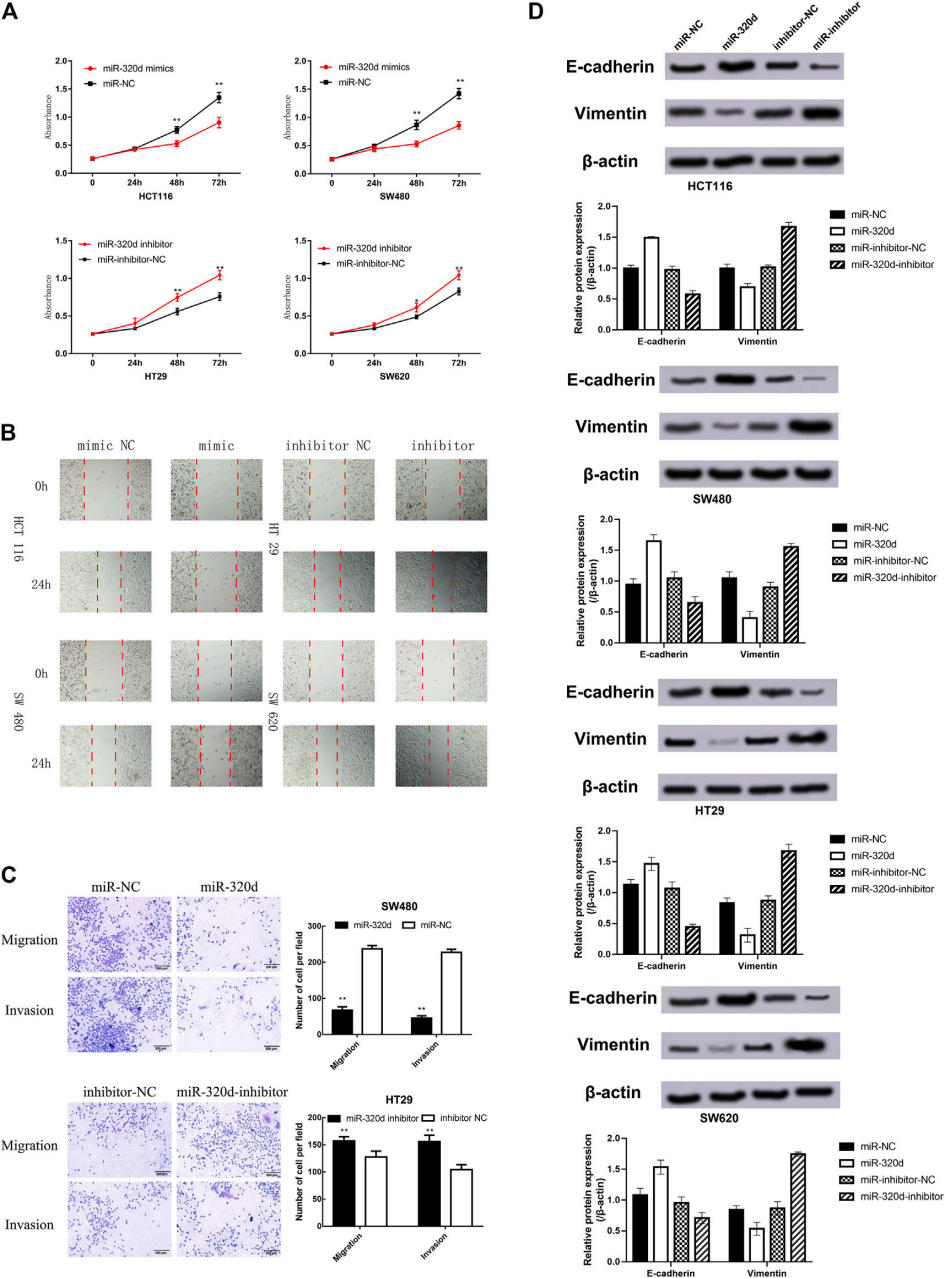
Ectopic expression of miR-320d inhibits the proliferation, invasion, and migration of EGFR-positive CRC cells. **(A)** MTT cell proliferation assays in EGFR-positive CRC cells transfected with indicated miRNAs. **(B)** Representative photomicrographs of wound-healing assay in EGFR-positive CRC cells after transfection of miRNAs. **(C)** Transwell assays of EGFR-positive CRC cells carrying different miRNAs. The total number of cells in the field was counted manually. Assays were performed in triplicates. Mean ± SD is shown. Statistical analysis was conducted using one-way ANOVA. **p< 0.01. **(D)** Western blots of E-cadherin and vimentin in EGFR-positive CRC cells transfected with different miRNAs.; Abbreviations: MTT, 3-(4,5-dimethylthiazol-2-yl)-2, 5-diphenyltetrazolium bromide; NC, negative control.

First, we examined the effect of miR-320d on the proliferation of CRC cells by MTT assay. The upregulation of miR-320d expression significantly decreased the proliferation ability of CRC cells, while miR-320d inhibitor-treated CRC cells showed a reversed phenotype (Figure 3A). Next, we examined the effect of miR-320d overexpression on the invasive ability of CRC cells. The wound-healing assay show that the miR-320d-overexpressed cells migrated toward the wound at a much slower rate than the cells treated by miR-320d inhibitors (Figure 3B). Transwell assays revealed that the cell invasion ability of the miR-320d overexpression group was significantly lower than that of the miR-320d downregulated group (Figure 3C), indicating that the upregulation of the miR-320d expression could significantly inhibit the invasion ability of CRC cells. Additionally, we explored the effects of miR-320d on the expressions of EMT markers in EGFR-positive CRC cells by using Western blotting. The overexpression of miR-320d in EGFR-positive CRC cells suppressed the mesenchymal marker vimentin and upregulated the epithelial marker E-cadherin (Figure 3D). Taken together, the above results highlight the ability of miR-320d in suppressing malignant phenotypes of EGFR-positive CRC cells.

### Tumor-Suppressor Candidate 3 Is a Direct Target of miR-320d

**FIGURE4:**
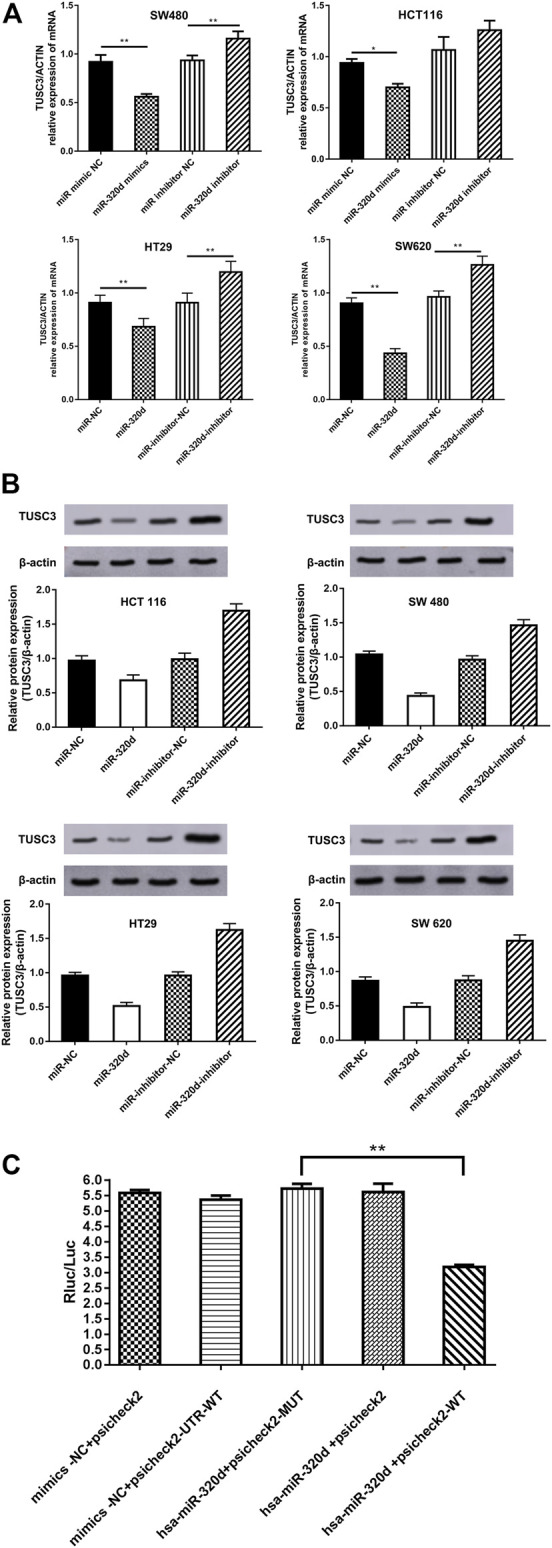
miR-320d targets the 3′-UTR of TUSC3 mRNA. **(A)** qRT-PCR analysis of TUSC3 mRNA expression in EGFR-positive CRC cells transfected with the indicated miRNAs. **(B)** Western blots of TUSC3 in EGFR-positive CRC cells transfected with different miRNAs. **(C)** Luciferase activity of reporter plasmids carrying wild-type or mutant TUSC3 3′-UTR in EGFR-positive CRC cells co-transfected with the indicated miRNAs. Assays were performed in triplicates. Mean ± SD is shown. Statistical analysis was conducted using Student’s t test. *p < 0.05. **p < 0.01.

To further confirm that the expressions of TUSC3 are indeed regulated by miR-320d in CRC cells, we transfected EGFR-positive CRC cells with miR-320d-mimic, miR-mimic-NC, miR-320d-inhibitor, and miR-inhibitor-NC. We confirmed that miR-320d decreased the mRNA levels of TUSC3 (Figure 4A) and inhibited the protein expressions (Figure 4B) in CRC cells, while the TUSC3 expression level was restored in miR-320d inhibitor-treated cells. The luciferase assays showed that miR-320d could significantly reduce the luciferase activities of the 3′UTR of TUSC3, but the luciferase activity was not significantly changed when TUSC3 3′UTR-binding sites are mutated (Figure 4C). These results suggest that miR-320d negatively regulates the TUSC3 expression.

### Overexpression of Tumor-Suppressor Candidate 3 Restores the Malignant Phenotype of Colorectal Cancer Cells

**FIGURE5:**
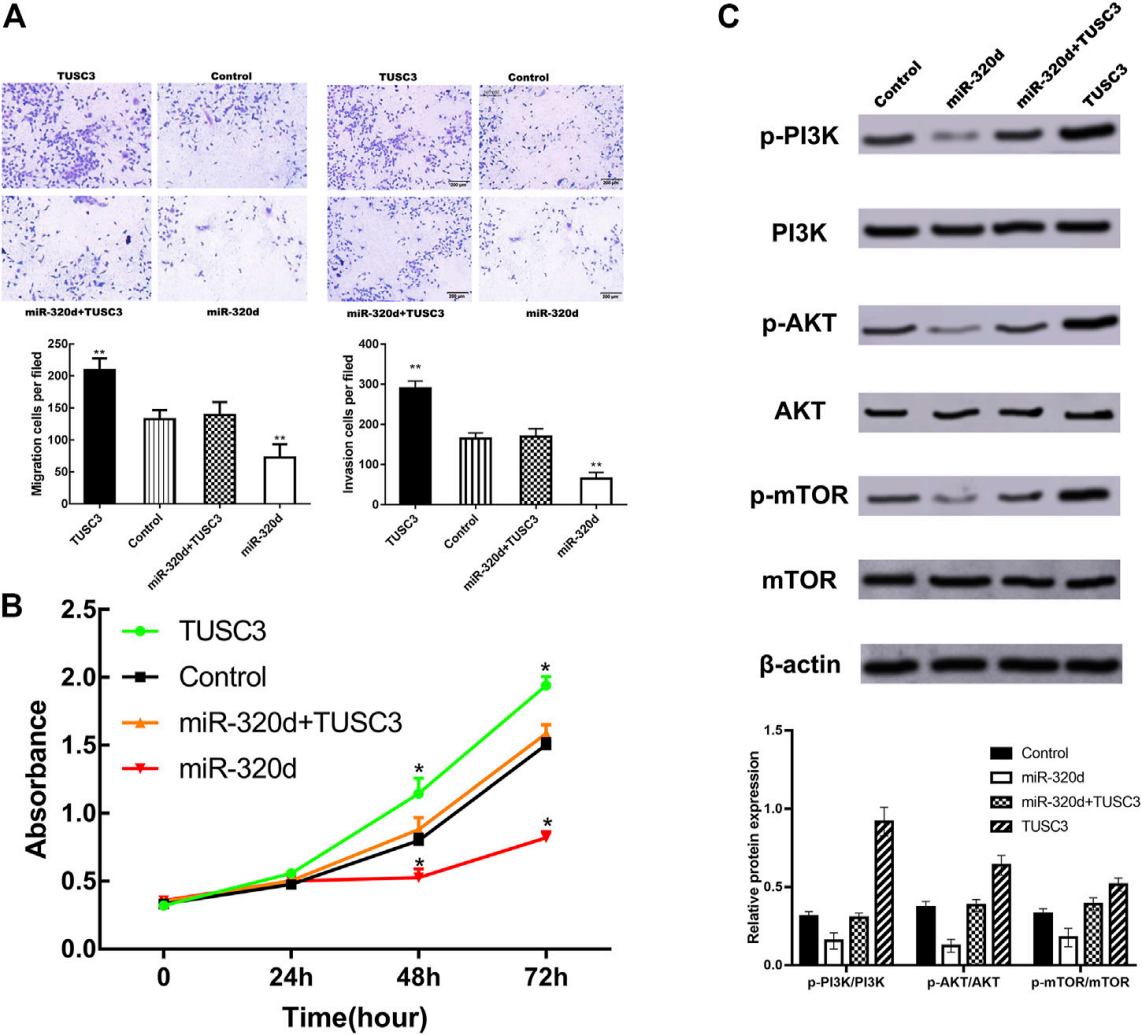
miR-320d inhibits the EGFR-positive CRC cell migration, invasion, and proliferation and negatively regulated PI3K/AKT/mTOR signaling pathway cells by downregulation of TUSC3. **(A)** Transwell assays and **(B)** MTT assays were conducted in EGFR-positive CRC cells transfected with miR-320d or miR-320d and TUSC3 or Control or TUSC3. **(C)** The expression of these proteins was detected by Western blot after the restoration of TUSC3. Assays were performed in triplicates. Mean ± SD is shown. Statistical analysis was conducted using one-way ANOVA. **p< 0.01; *p< 0.05, compared to miR-320d and TUSC3 groups.; Abbreviations list.

We performed the rescue experiments by co-transfecting EGFR-positive CRC cells with miR-320d mimics and TUSC3 overexpression plasmid to observe the changes in biological functions of CRC cells. Overexpression of TUSC3 largely reversed the inhibitory effect of miR-320d on invasion (Figure 5A) and proliferation (Figure 5B) of CRC cells. These results suggest that miR-320d inhibits the malignant phenotype of EGFR-positive CRC cell which is mediated by TUSC3.

### miR-320d Inhibits the PI3K/AKT Signaling Pathway in Colorectal Cancer

Combined with the results of bioinformatics analysis of this study, we further explored the effects of miR-320d on the classic PI3K/AKT/mTOR signaling pathways. We found that the expression of p-PI3K, p-AKT, and p-mTOR was significantly decreased in EGFR-positive CRC cells transfected with miR-320d-mimics. In addition, this effect was partially blocked by upregulation of TUSC3 expression in CRC cells (Figure 5C). These data suggest that the PI3K/AKT/mTOR signaling pathway may mediate the function of miR-320d in EGFR-positive CRC cells.

## Discussion

The target genes of many miRNAs are proto-oncogenes or tumor suppressor genes, and the abnormal expression of miRNAs is a common feature of tumorigenesis (Al-Akhrass and Christou, 2021). We hope that the microRNA microarray can help us find a potential therapeutic approach for EGFR-positive CRC. In our study, we used CRC microRNA microarray data and screened for differentially expressed miRNAs in EGFR-positive CRC. Combined with literature search and bioinformatics analysis, miR-320d caught our attention. We examined miR-320d expression in EGFR-positive CRC and the paired adjacent normal tissue and found that its expression was significantly downregulated in EGFR-positive CRC. Then, we investigated the clinical significance of miR-320d in EGFR-positive CRC and examined its role in the proliferation and invasion of EGFR-positive CRC cells. We found that miR-320d expression was significantly lower in EGFR-positive CRC cells, and overexpression of miR-320d inhibited the proliferation and invasion of EGFR-ositive CRC cells. Therefore, miR-320d may function as a tumor suppressor in EGFR-positive CRC. miR-320d, a member of the miRNA-320 family, is expressed at very low levels in many human malignancies and has a tumor-suppressive effect (Li et al., 2021). Prior studies have noted the importance of the microRNA-320 family in colorectal adenoma. However, previously published studies on the effect of miR-320d in CRC are not consistent, and qRT-PCR analyses showed a decreasing expression of the miR-320 family, except miR-320d, from non-neoplastic mucosa through adenoma to submucosal invasive carcinoma (Tadano et al., 2016). Further, the carcinogenic mechanisms of miR-320d were poorly understood, particularly in EGFR-positive CRC. Our study seems to be consistent with other gastrointestinal cancer research which found that miR-320d was downregulated in gastric cardiac adenocarcinoma tissues, and patients with a lower miR-320d expression had a worse prognosis compared to those with a higher miR-320d expression (Chen et al., 2020). A strong relationship between miR-320d and the hepatocellular carcinoma malignant phenotype has been reported in the literature and overexpression of miR-320d inhibited the proliferation and invasion of hepatocellular carcinoma cells (Li et al., 2020). In our study, the overexpression of miR-320d suppressed the EGFR-positive CRC cell proliferation and invasion. These results corroborate the ideas above the article, which suggested that miR-320d may function as a tumor suppressor.

Tumor-suppressor candidate 3 (TUSC3), also called Oligosaccharyltransferase 3 Homolog A, is closely related to tumor development (Chung et al., 2012). Studies on ovarian cancer cell lines and tumor specimens showed that the TUSC3 expression was significantly downregulated in ovarian cancer due to hypermethylation of the TUSC3 promoter region, and deletion of TUSC3 could promote proliferation and metastasis of ovarian cancer cells (Pils et al., 2013). Another study showed that TUSC3 expression was downregulated in small cell lung cancer, and TUCS3 may become a predictor of lymph node metastasis in lung cancer patients (Yu et al., 2016). Previous studies have shown that TUSC3 is related to CRC prognosis (Zhu and Dong, 2018). However, the correlation of TUSC3 with miR-320d in EGFR-positive CRC is still unknown. In our study, miR-320d transfection significantly reduced the mRNA and protein expression levels of TUSC3 in EGFR-positive CRC cells. We confirmed that miR-320d targets the 3′-UTR of TUSC3 by a dual luciferase gene reporter assay, and the recovery of TUSC3 partially counteracts the effects of the proliferation, migration, and invasion of CRC cells by miR-320d mimics. Our study showed that miR-320d target genes were mainly enriched in the PI3K/Akt pathway, and miR-320d overexpression decreased p-AKT, p-PI3K, and p-mTOR. These data suggest that miR-320d suppresses the malignant biology behavior of EGFR-positive CRC cells by partially inhibiting PI3K/Akt/mTOR signaling pathways. In addition, we downloaded CRC data in the TCGA database and classified them into CRC tumors with high and low TUSC3 expressions. GSEA analysis showed a strong correlation between TUSC3 expression and the EMT signaling pathway. EMT is the conversion of epithelial cells into mesenchymal cells and the acquisition of invasive capacity, leading to cell metastasis. Based on this result, we further verified whether miR-320d is involved in the process of EMT in CRC. Vimentin was significantly decreased, and E-cadherin was significantly increased after transfection of the miR-320d mimic in EGFR-positive CRC cells; therefore, we inferred that miR-320d is an important regulatory miRNA that can inhibit the EMT process.

## Conclusion

In conclusion, our research identified a novel tumor suppressor in EGFR-positive CRC and elucidated its mechanism. Our research demonstrated that miR-320d could inhibit proliferation, migration, invasion, and EMT by inhibiting TUSC3, suggesting that miR-320d may be applied as a key therapeutic target for clinical treatment, providing a new strategy for the prevention and treatment of EGFR-positive CRC.

## Data Availability

The datasets presented in this study can be found in online repositories. The names of the repository/repositories and accession number(s) can be found below: https://www.ncbi.nlm.nih.gov/geo/, GSE62007.
